# Use of Cidofovir for Safe Transplantation in a Toddler with Acute Liver Failure and Adenovirus Viremia

**DOI:** 10.1155/2022/9426175

**Published:** 2022-11-09

**Authors:** Vikram J. Christian, Raiya Sarwar, Joseph C. Resch, Sarah Lim, Arif Somani, Catherine Larson-Nath, Shane McAllister, Beth K. Thielen, Oyedele Adeyi, Srinath Chinnakotla, Heli Bhatt

**Affiliations:** ^1^Department of Pediatrics, Division of Pediatric Gastroenterology, Hepatology, and Nutrition, University of Minnesota Masonic Children's Hospital, Minneapolis, Minnesota, USA; ^2^Department of Medicine, Division of Transplant Hepatology, University of Minnesota, Minneapolis, Minnesota, USA; ^3^Department of Pediatrics, Division of Pediatric Critical Care Medicine, University of Minnesota Masonic Children's Hospital, Minneapolis, Minnesota, USA; ^4^Minnesota Department of Health, St. Paul, Minnesota, USA; ^5^Department of Pediatrics, Division of Pediatric Infectious Diseases, University of Minnesota Masonic Children's Hospital, Minneapolis, Minnesota, USA; ^6^Department of Laboratory Medicine and Pathology, University of Minnesota, Minneapolis, Minnesota, USA; ^7^Department of Surgery, Division of Transplant Surgery, University of Minnesota, Minneapolis, Minnesota, USA

## Abstract

**Background:**

Since October 2021, there have been more than 500 cases of severe hepatitis of unknown origin in children reported worldwide, including 180 cases in the U.S. The most frequently detected potential pathogen to date has been adenovirus, typically serotype 41. Adenovirus is known to cause a self-limited infection in the immunocompetent host. However, in immunosuppressed individuals, severe or disseminated infections may occur.

**Method:**

We present the case of a two-year-old female who presented with cholestatic hepatitis and acute liver failure (ALF). Work up for etiologies of ALF was significant for adenovirus viremia, but liver biopsy was consistently negative for the virus. The risk for severe adenoviral infection in the setting of anticipated immunosuppression prompted us to initiate cidofovir to decrease viral load prior to undergoing liver transplantation.

**Result:**

Our patient received a successful liver transplant, cleared the viremia after 5 doses of cidofovir, and continues to maintain allograft function without signs of infection at the time of this report, 5 months posttransplant.

**Conclusion:**

Recent reports of pediatric hepatitis cases may be associated with adenoviral infection although the exact relationship is unclear. There is the possibility of the ongoing SARS-CoV-2 environment, or other immunologic modifying factors. All patients presenting with hepatitis or acute liver failure should be screened for adenovirus and reported to state health departments. Cidofovir may be used to decrease viral load prior to liver transplantation, to decrease risk of severe adenoviral infection.

## 1. Introduction

The World Health Organization (WHO) on April 15, 2022, reported a spike in pediatric hepatitis of unknown etiology (74 cases) across the United Kingdom [1]. On April 29, 2022, the Center for Disease Control (CDC) in its Morbidity and Mortality Weekly Report detailed a series of pediatric hepatitis cases in Alabama, some of which had progressed to acute liver failure (ALF) requiring liver transplantation. The 9 patients with hepatitis (median age 2.9 years) experienced vomiting, diarrhea, and upper respiratory symptoms, and on admission were found to have jaundice/scleral icterus, hepatomegaly and/or encephalopathy. Adenovirus was detected in blood by PCR in all 9 patients. Three of these patients developed ALF, two of whom were treated with cidofovir and steroids, and underwent liver transplantation [2]. Marsh et al. of Scotland reported 13 cases of hepatitis (median age 3.9 years) who presented with jaundice, abdominal pain, nausea, and malaise, with one patient undergoing liver transplantation. Five of these 13 patients tested positive for adenovirus [3].

The WHO multicountry report published on April 23, 2022 [4], detailed 169 cases of acute hepatitis across 11 countries. Of these patients, aged between 1 month and 16 years, 17 required liver transplantation and 1 death was reported. Adenovirus was detected in 74 cases, 18 of which have been identified as serotype F type 41. SARS-CoV-2 was detected in 20 cases of those that were tested. Coinfection of SARS-CoV-2 and adenovirus was found in 19 patients.

On June 14, 2022, the CDC reported that, in comparison to a pre–COVID-19 pandemic baseline, there has not been a significant increase in weekly ED visits with hepatitis-associated discharge codes, number of hepatitis-associated hospitalizations, or number of monthly liver transplants during October 2021–March 2022 among children aged 0–4 or 5–11 years. It was also reported that the percentage of specimens positive for adenovirus types 40/41 among children aged 0 to 4 and 5 to 9 years did not appear to increase above prepandemic historical levels. The CDC recommends continued surveillance to monitor trends over time [5].

Although adenovirus has been identified in some of these patients presenting with hepatitis/ALF, to date no definitive etiology for these cases of hepatitis is known [6]. Adenovirus is considered a common pathogen, with worldwide distribution. Adenovirus is known to cause a self-limited infection in the immunocompetent host. However, severe or disseminated infections may occur in immunosuppressed individuals with history of previous solid organ transplant, hematopoietic stem cell transplant, or hematological malignancy [7].

We present the case of a two-year old female who presented with cholestatic hepatitis and acute liver failure. Work up for etiologies of ALF was significant for adenovirus viremia. The risk for severe adenoviral infection in the setting of anticipated immunosuppression prompted us to initiate cidofovir to decrease viral load prior to undergoing liver transplantation.

## 2. Case Description

Our patient is a 2-year-old fully vaccinated female whose past medical history includes neonatal hyperbilirubinemia treated with prolonged outpatient phototherapy, multiple episodes of acute otitis media, and peanut allergy. She presented with pale stools, dark urine, jaundice, scleral icterus, loss of appetite, and irritability. At presentation, she was on day 4 of oral amoxicillin therapy for otitis media. In the prior week, she was also seen for hives without a confirmed peanut exposure. She was treated with epinephrine and did not require hospitalization. She was not exposed to any other over-the-counter or prescribed medications (including acetaminophen), herbs, supplements, or pesticides. Family history was notable for recent sudden unexplained infant death in an 11-month-old sibling and reports of a distant cousin needing liver transplant in childhood. In addition, immediate family members had SARS-CoV-2 in the months prior to presentation.

At presentation, patient had reassuring vital signs and was appropriately fussy for age. Exam was notable for firm hepatomegaly, jaundice, a fine exanthematous rash noted upon her extremities and trunk, II-III/VI flow murmur, and mild delayed capillary refill. Pertinent initial laboratory data included elevated total and direct bilirubin at 9.8 mg/dL and 6.5 mg/dL, respectively, AST 7040 U/L, ALT > 5000 U/L, alkaline phosphatase 464 U/L, gamma-glutamyl transferase (GGT) 167 U/L, INR of 2.08, and serum lactic acid 3.9 mmol/L.

Following admission to our pediatric intensive care unit (PICU), she underwent extensive diagnostic work up in pursuit of an etiology for hepatic failure (Table 1). PCR on blood was positive for adenovirus at log 4.3 copies/mL (serotyping pending). Her preadmission SARS-CoV-2 PCR was negative, but SARS-CoV-2 serology testing showed detectable antispike and antinucleocapsid IgG. A respiratory pathogen panel PCR was positive for rhinovirus/enterovirus. EBV DNA was detectable in whole blood at log 3.4 copies/mL but not in plasma, and serologies were consistent with past infection. Anti-CMV IgG was positive, but IgM and DNA PCR were negative. There was no evidence of metabolic or space-occupying etiology identified. Our patient did have elevated soluble Interleukin 2 Receptor Subunit Alpha but normal natural killer (NK) cell function. Preliminary results of hereditary whole exome sequencing demonstrated one maternally inherited pathogenic variant in SERPINA1 gene.

**Figure 1:**
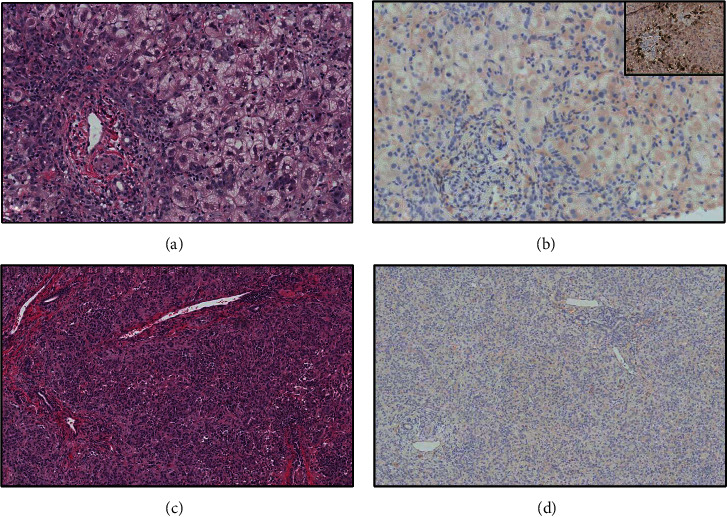
Liver biopsy (a, b) and explanted liver (c, d) show marked hepatitis that demonstrates progression from the biopsy time point to the explant. Panel a (Hematoxylin and Eosin) shows marked hepatitis with swollen hepatocytes, compared to the explant (panel c, Hematoxylin and Eosin), where most of the swollen hepatocytes are no longer visible while active inflammation persists. In both samples, adenovirus immunohistochemistry was negative (panels b and d; inset shows positive control for adenovirus). (Original magnification, panels a–d: 100×).

Liver biopsy was performed on day of admission (DOA) 2, showing severe diffuse hepatocellular swelling with moderate portal and lobular lymphocytic and neutrophilic infiltration/inflammation without fibrosis (Figure 1). Immunostaining was negative for adenovirus and CMV. Copper stains showed no evidence of copper retention. In situ hybridization using Epstein-Barr encoded RNA probes was also negative for EBV.

Her PICU course included progressive hypoglycemia requiring intravenous supplementation with high dextrose containing fluids, parental nutrition, and intermittent feeds when feasible and tolerated. Her hemodynamics and respiratory status were unremarkable. She had robust urine output with no evidence of acute kidney injury. She developed worsening coagulopathy that was not reversible with vitamin K and was listed for liver transplantation as status 1A on DOA 3 due to progressive liver failure. Irritability worsened throughout her initial hospital course, consistent with early grade I encephalopathy. Her ammonia level remained relatively low (<50 umol/L) likely facilitated by her hyposthenic body habitus and enteral lactulose administration. By DOA 9, she developed altered mental status alongside a rising ammonia level (>100 umol/L) and continuous renal replacement therapy was initiated.

Cidofovir was initiated prior to transplant to decrease the risk of ongoing viral replication on the grafted liver. Usage of cidofovir was consistent with the care of two patients in the Alabama cohort who proceeded to transplant [2]. Initial therapy consisted of 1 mg/kg intravenous cidofovir (on DOA 8) and 12.5 mg/kg enteral probenecid with fluid bolus every 48 hours. This regimen was used to minimize cidofovir-associated nephrotoxicity in the setting of acute liver failure. Our patient received one dose of cidofovir and intravenous immunoglobulin (IVIG) prior to transplant, which dropped her adenovirus titer from a peak of log 4.3 to log 4. Serology testing to identify an etiology was completed prior to IVIG administration. Cidofovir was stopped after five doses, when adenovirus was no longer detectable in blood. Adenovirus PCR remained negative one week after cidofovir was discontinued. No evidence of kidney injury developed during our reporting period.

She underwent deceased donor ABO compatible reduced liver transplantation on DOA 10 using left lateral segment (segment 2 and 3) with a graft weight to recipient weight ratio of 3.6%. She required an aortohepatic conduit as the native artery was diminutive, and a staged closure with a biological mesh due to large for size liver graft. She did not require any intra- or postoperative dialysis. Immunosuppression regimen included induction with basiliximab and methylprednisolone followed by maintenance with mycophenolate and later, tacrolimus. The explanted liver showed progressive hepatocellular damage compared with pretransplant biopsy but still stained negative for adenovirus, CMV, and EBV. Alprostadil infusion was started later. Her coagulopathy corrected, she demonstrated adequate hepatic and renal function, and remains free of infection at the time of this report, 5 months posttransplant.

## 3. Discussion

Since October 2021, more than 500 cases of severe hepatitis of unknown origin in children have been reported worldwide, including 180 cases in the U.S. [8, 9] Most cases have occurred in healthy children under the age of 5, and no epidemiological links between cases and common environmental exposures have been identified. The most frequently detected potential pathogen to date has been adenovirus, typically serotype 41, with 50-60% of children in the U.K. and U.S. testing positive, primarily in blood [10].

Adenoviruses typically cause respiratory or gastrointestinal illness in children, and infections occur throughout the year without seasonality. Outbreaks have been reported in the community or in close-contact settings, such as daycare centers, schools, summer camps, and in military recruits. Adenovirus type 41 is uncommon in the U.S., accounting for only 0.3% of human adenovirus detections on national surveillance from 2003 to 2016 [11]. While adenovirus serotype 41 has been associated with infantile gastroenteritis, it has not been typically associated with hepatitis until now, especially in immunocompetent children.

There is yet an unclear role for adenovirus in our patient's hepatitis, although several hypotheses have been proposed in trying to explain these new pediatric hepatitis cases. One is a lack of exposure to routinely circulating adenovirus strains due to isolation requirements during the COVID-19 pandemic, causing decreased immunity in younger children and a subsequent increase in more severe disease manifestations [12]. Another possibility is an increased overall incidence in adenovirus infections following relaxation of pandemic restrictions, resulting in unmasking of a previously unrecognized rare manifestation of adenovirus infection in healthy children [13–15]. Also proposed is the likely emergence of a new strain of adenovirus with increased tendency to cause this type of severe hepatitis.

The lack of direct evidence of adenovirus on immunostaining of liver tissue points towards a possible virus-induced hyperimmunologic reaction rather than direct viral cytotoxicity, but the specific pathogenesis of liver injury remains unknown. Cytokine storm has been identified as a likely major factor in COVID-19 infected patients who developed liver failure [16]. The role of SARS-CoV-2 as a potential cofactor remains an important clinical focus, either as a postinfectious sequela like multisystem inflammatory syndrome in children, or potentially due to SARS-CoV-2 superantigen-mediated immune activation [17]. SARS-CoV-2 environment could have played a role in adenovirus-driven acute liver injury in this patient.

Adenovirus infection in the setting of liver transplantation ranges from clinically silent to fulminant disseminated disease. In a series of 6 asymptomatic pediatric patients with history of solid organ or stem cell transplantation who were found to have adenovirus detected in blood (by PCR), 2 patients remained asymptomatic, 1 developed hemorrhagic enteritis and cystitis. Three of the 6 patients developed high adenoviral load, clinical sepsis, and died of multiorgan failure despite antiviral therapy with cidofovir [18]. A retrospective review of 484 immunosuppressed pediatric liver transplant recipients revealed that among the 49 patients with posttransplant adenoviral infection, 20 had invasive infection, resulting in the death of 9 patients [19]. Given the high frequency of asymptomatic adenovirus viremia, liver transplant recipients are not routinely screened [20].

Currently, there is a considerable uncertainty about the role of adenovirus in cases of acute liver failure in children, and there are no published guidelines on the use of cidofovir in these cases. While there are concerns about transplanting a child with active adenovirus viremia, the IVIG coupled with five doses of cidofovir was helpful in decreasing the viral load in our patient. Cidofovir is known to cause nephrotoxicity [21]. The use of tacrolimus posttransplant can further worsen this problem [22]. Cidofovir was well tolerated in our patient without nephrotoxicity. Probenecid and prostaglandin infusion were used in our patient and perhaps afforded renal protection [23, 24].

## 4. Conclusion

Recent reports of pediatric hepatitis cases may be associated with adenoviral infection although the exact relationship is unclear, and other modifying factors may be important. All patients presenting with hepatitis or ALF should be screened for adenovirus and reported to state health departments. Cidofovir may be used safely to decrease viral load prior to liver transplantation, as well as in peritransplant and immediate posttransplant period to decrease risk of severe adenoviral infection post liver transplant.

## Figures and Tables

**Table 1:** Diagnostic work up.

Diagnostic work up
*Infectious* Blood culture, **respiratory viral panel**, NP chlamydia pneumonia PCR, NP mycoplasma PCR, hepatitis A Ab, hepatitis B surface Ab, hepatitis B surface Ag, hepatitis B core Ab, hepatitis B e Ag, hepatitis B DNA serum quantitative assay, hepatitis C Ab, hepatitis C RNA quantitative assay, EBV capsid IgM and **IgG**, EBV nuclear Ab, **EBV early IgG**, **EBV serum quantitative assay whole blood**, EBV quantitative assay plasma, CMV IgM and **IgG**, CMV DNA serum quantitative assay, HIV 1/2 Ag and Ab, HHV6 serum PCR, enterovirus serum PCR, **adenovirus serum PCR**, **adenovirus serum quantitative assay**, adenovirus stool PCR, *Parechovirus* serum PCR, Quantiferon-tuberculosis gold, SARS-CoV-2 PCR, **COVID-19 spike Ab**, **COVID-19 nucleocapsid Ab**
*Hepatic* **AST**, **ALT**, **albumin**, **alkaline phosphatase**, **lactate**, **GGT**, **lactate dehydrogenase**, **total bilirubin**, **direct bilirubin**, ammonia, glucose, amylase, lipase, **factor V**, **factor VII**, factor VIII, alpha-fetoprotein, abdominal ultrasound, **liver biopsy**
*Metabolic* **Ferritin**, **iron studies**, **pyruvic acid**, acylcarnitine panel, **lipid profile**, **alpha-1-antitrypsin phenotype**, alpha-1-antitrypsin level, **vitamin A level**, **vitamin D level**, vitamin E level
*Immunologic* Coombs test, antinuclear antibody, tissue transglutaminase Ab, **IgG level**, **immunoglobulin A level**, NK cell function, liver-kidney-microsomal Ab, **F-actin (smooth muscle) Ab level**, **F-actin (smooth muscle) Ab titer**, varicella IgG, rubeola IgG, rubella IgG, mumps IgG, **soluble interleukin 2 receptor subunit alpha**, NK cell subset
*Hematologic* Complete blood count, **INR**, partial thromboplastin time, **fibrinogen**, **D-dimer**, haptoglobin, **thromboelastography**, type & screen, **blood pathology**, RBC Ab titer
*Electrolyte/renal* Basic metabolic panel, magnesium, **phosphorous**, creatine kinase, uric acid, urinalysis
*Cardiac* Echocardiogram, electrocardiogram
*Genetic* **Whole exome sequencing (preliminary results)–pathogenic variant in SERPINA1 gene**

^∗^Abnormal values on initial checks are **bolded**. Positive values for vaccination checks were not marked as abnormal.

## Abbreviations

Ab: Antibody
Ag: Antigen
ALF: Acute liver failure
ALT: Alanine transaminase
AST: Aspartate transaminase
CMV: Cytomegalovirus
COVID-19: Coronavirus disease 19
DNA: Deoxyribonucleic acid
DOA: Day of admission
EBV: Epstein-Barr virus
GGT: Gamma-glutamyl transferase
HBV: Hepatitis B virus
HCV: Hepatitis C virus
HHV6: Human herpesvirus 6
IgG: Immunoglobulin G
IgM: Immunoglobulin M
INR: International normalized ratio
IVIG: Intravenous immunoglobulin
NK: Natural killer
NP: Nasopharyngeal
PCR: Polymerase chain reaction
PICU: Pediatric intensive care unit
SARS-CoV-2: Severe acute respiratory syndrome coronavirus 2.
